# Unravelling the Diversity of the Cyclopiazonic Acid Family of Mycotoxins in *Aspergillus flavus* by UHPLC Triple-TOF HRMS

**DOI:** 10.3390/toxins9010035

**Published:** 2017-01-13

**Authors:** Valdet Uka, Geromy G. Moore, Natalia Arroyo-Manzanares, Dashnor Nebija, Sarah De Saeger, José Diana Di Mavungu

**Affiliations:** 1 Laboratory of Food Analysis, Faculty of Pharmaceutical Sciences, Ghent University, Ottergemsesteenweg 460, 9000 Ghent, Belgium; valdet.uka@ugent.be (V.U.); natalia.arroyomanzanares@ugent.be (N.A.-M.); sarah.desaeger@ugent.be (S.D.S.); 2 Department of Pharmacy, Faculty of Medicine, University of Prishtina, Rrethi i Spitalit p.n, 10000 Prishtina, Kosovo; dashnor.nebija@uni-pr.edu; 3 Southern Regional Research Center, Agricultural Research Service, United States Department of Agriculture (ARS-USDA), New Orleans, 70124 LA, USA; geromy.moore@ars.usda.gov; 4 Department of Analytical Chemistry, Faculty of Sciences, University of Granada, Campus Fuentenueva s/n, E-18071 Granada, Spain

**Keywords:** cyclopiazonic acid, ergot-like alkaloid, dereplication, HRMS

## Abstract

Cyclopiazonic acid (α-cyclopiazonic acid, α-CPA) is an indole-hydrindane-tetramic acid neurotoxin produced by various fungal species, including the notorious food and feed contaminant *Aspergillus flavus*. Despite its discovery in *A. flavus* cultures approximately 40 years ago, its contribution to the *A. flavus* mycotoxin burden is consistently minimized by our focus on the more potent carcinogenic aflatoxins also produced by this fungus. Here, we report the screening and identification of several CPA-type alkaloids not previously found in *A. flavus* cultures. Our identifications of these CPA-type alkaloids are based on a dereplication strategy involving accurate mass high resolution mass spectrometry data and a careful study of the α-CPA fragmentation pattern. In total, 22 CPA-type alkaloids were identified in extracts from the *A. flavus* strains examined. Of these metabolites, 13 have been previously reported in other fungi, though this is the first report of their existence in *A. flavus*. Two of our metabolite discoveries, 11,12-dehydro α-CPA and 3-hydroxy-2-oxo CPA, have never been reported for any organism. The conspicuous presence of CPA and its numerous derivatives in *A. flavus* cultures raises concerns about the long-term and cumulative toxicological effects of these fungal secondary metabolites and their contributions to the entire *A. flavus* mycotoxin problem.

## 1. Introduction

The ergot-like alkaloid cyclopiazonic acid (α-cyclopiazonic acid, α-CPA) is an indole-hydrindane-tetramic acid mycotoxin produced by many fungal species in the Ascomycete genera *Penicillium* and *Aspergillus*. α-CPA was first isolated from a liquid culture of *Penicillium cyclopium* Westling in 1968, as the main toxic compound of this microorganism [1]. Afterwards, in 1973, Ohmomo et al. [2] reported its production by a strain of *Aspergillus versicolor*. In 1977, it was also demonstrated that α-CPA can be produced by *Aspergillus flavus*, a prolific food and feed contaminant [3]. Since then, CPA-producing strains have been identified in other fungal species such as *Penicillium griseofulvum*, *Penicillium commune*, *Penicillium chrysogenum*, *Aspergillus oryzae*, *Aspergillus fumigatus* and *Aspergillus tamarii* [4,5,6,7].

A gene cluster for the biosynthesis of α-CPA, containing three essential genes, was identified in the genome of *A. flavus* and *A. oryzae*, situated adjacent to the aflatoxin gene cluster [8,9]. α-CPA is biosynthesized from three precursors including a tryptophan residue, two units of acetic acid and an isoprenoid moiety (dimethylallyl diphosphate—DMAPP) in a three-enzyme biochemical pathway. Through this short metabolic pathway, two biosynthetic intermediates are generated, *cyclo*-acetoacetyl-l-tryptophan (cAATrp) and β-cyclopiazonic acid (β-CPA), by consecutive action of three enzymes, CpaS, CpaD and CpaO. The hybrid two-module polyketide synthase-nonribosomal peptide synthetase (PKS-NRPS), i.e., CpaS (also known as *CpaA*), is responsible for the formation of the tetramic acid *cyclo*-acetoacetyl-L-tryptophan (cAATrp). cAATrp is then prenylated by the prenyltransferase CpaD (also known as *dmaT*), which leads to the generation of β-CPA, the ultimate tricyclic precursor of α-CPA. The final conversion of β- to α-CPA is catalyzed by the putative monoamine cyclo-oxidase CpaO (also known as *maoA*) in a redox reaction forming two rings (ring C and D) [10,11].

The biochemical mechanism beyond the toxicological profile of α-CPA is proved to be its specific ability to inhibit sarco/endoplasmic reticulum Ca^2+^-ATPase (SERCA) in different tissues and cell types [4,12]. Hence, α-CPA is considered one of the few potent, selective and reversible SERCA inhibitors, another example being thapsigargin [13]. SERCA is an active membrane pump responsible for the transfer of Ca^2+^ ions from the cytosol of the cell to the lumen of the sarco/endoplasmic reticulum, thus maintaining a low concentration of free calcium ions in the cytosol. Physiologically, these levels of intracellular calcium are essential for housekeeping activities of each cellular entity, like cell signaling, proliferation, differentiation and muscle contraction-relaxation. For this reason, SERCA blockage by α-CPA disrupts the normal intracellular calcium gradient and eventually leads to cell damage and death. Although the main target organs of CPA poisoning seem to be skeletal muscle, hepatic tissues and spleen, several animal studies have also reported pathological lesions in the kidney, pancreas, heart and gastro-intestinal tract [14,15]. Moreover, it has been demonstrated that CPA exposure can be associated with several neurological symptoms like hypokinesia, catalepsy, hypothermia, tremors, convulsions, cessation of food intake and resulting cachexia [16]. In general, α-CPA is not considered an acute mycotoxin due to its relatively high LD_50_ ranges in rats (30–70 mg/kg) and because of the benign nature of the intoxication [17]. Although confirmed CPA-mycotoxicoses have not been reported in humans, it was speculated that CPA exposure may be linked with “kodo poisoning,” a toxic syndrome characterized by nausea, vomiting, depression and unconsciousness after intake of CPA-contaminated Kodo millet [18].

The risk of human exposure to α-CPA could arise from the consumption of contaminated food commodities. α-CPA has been found to contaminate various grains and seeds, as well as different food matrices such as cheese, nuts and meat products [19,20,21,22]. Furthermore, this mycotoxin has been reported in milk and eggs, most likely due to animal consumption of contaminated feeds [23]. The importance of α-CPA as a toxic contaminant of different foodstuffs has been neglected and masked by other concurrent mycotoxins such as aflatoxins, especially when co-occurrence of CPA and aflatoxins has been observed [24,25,26,27,28].

**Figure 1:**
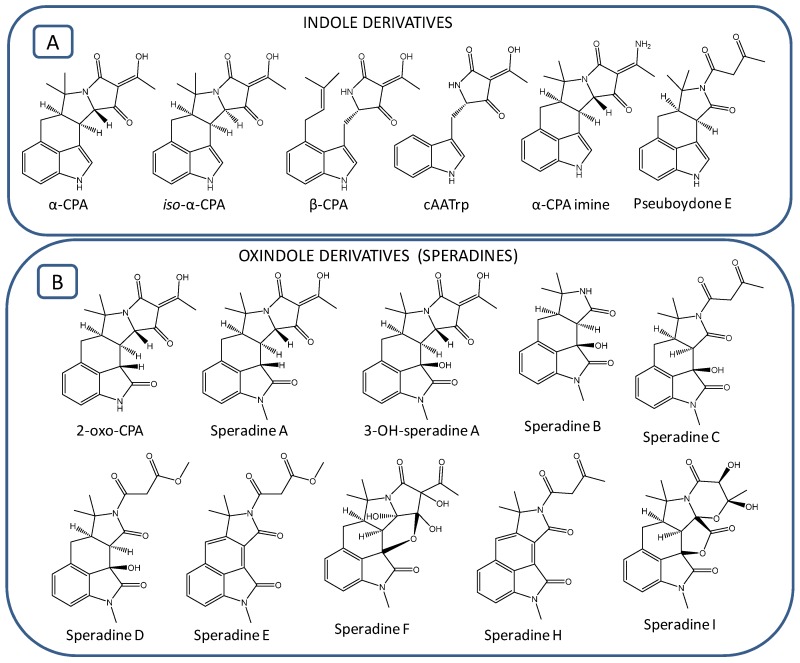

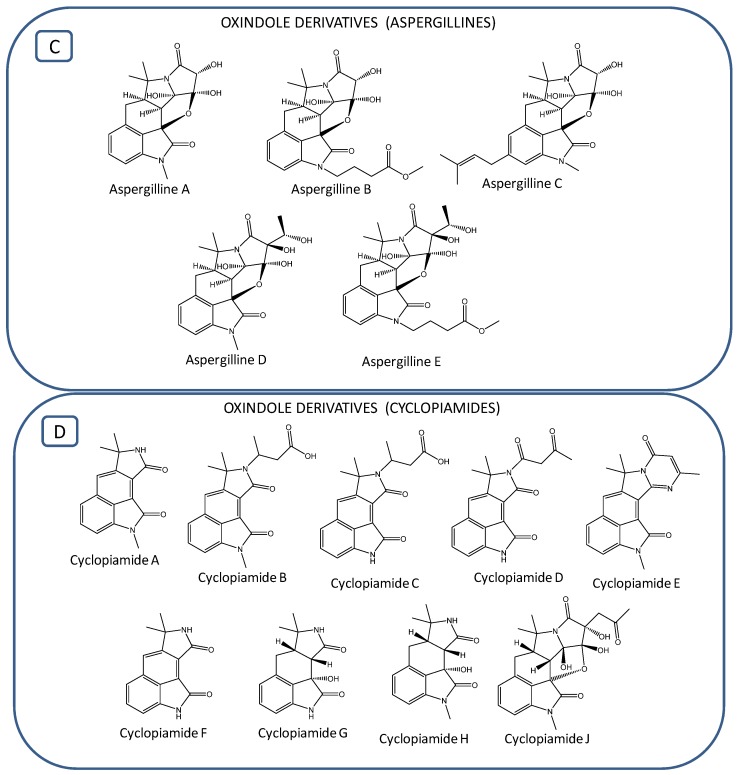
Structures of cyclopiazonic acid (CPA)-type alkaloids: (**A**) Indole derivatives; (**B**) Speradines; (**C**) Aspergillines; (**D**) Cyclopiamides.

Based on our literature review, since 1968 when α-CPA was first isolated, approximately 30 cyclopiazonic acid (CPA)-type alkaloids have been reported in different fungal extracts of aspergilli and penicillium (Figure 1; Table 1). These naturally occurring CPA analogues, containing the tetramate moiety as key structural motif, are characterized by some minor structural variations, and all belong to indole or oxindole (indolinone) subclasses of alkaloids. Very soon after the α-CPA discovery, Holzapfel et al. [29] reported the identification of two other CPA derivatives: β-CPA (also termed *bis*-secodehydrocyclopiazonic acid) and α-CPA imine. Later on, *iso*-α-CPA, the isomer of α-CPA, was structurally characterized in *A. flavus* [30]. Very recently, a new CPA derivative, pseuboydone E, has been isolated in *Pseudallescheria boydii* [31].The above-mentioned metabolites, namely α-CPA, *iso*-α-CPA, β-CPA, α-CPA imine, cAATrp and pseuboydone E, are the only CPA derivatives belonging to the indole subclass of CPA-type alkaloids (Figure 1A). All the remaining analogues, i.e., 2-oxoCPA, speradines, cyclopiamides and aspergillines, are classified in the oxindole subclass of CPA-type alkaloids. The first member of oxindoles possessing a keto-group in the C2-position of the indole nucleus is 2-oxoCPA; its presence has been reported in *A. oryzae* [32,33]. The first N-methylated pentacyclic oxindole analogues of α-CPA, speradine A and 3-hydroxyl-speradine A were isolated in fungal cultures of *A. tamarii* [34,35]. Four other tetracyclic oxindole alkaloids, named speradine B, C, D and E, were identified from *A. oryzae* [36]. A rare hexacyclic oxindole alkaloid, speradine F (also termed penicamedine A), together with two novel tetracyclic oxindoles, speradine G and H, were characterized in *A. oryzae* isolated from river sediments in China [37,38] (Figure 1B). In the literature, the nomenclature of oxindoles has been utilized incorrectly. For example, Ma et al. [39] reported the identification of speradine B, C and D from a sponge-derived strain of *A. flavus*, but these molecules do not correspond with metabolites previously described by Hu and coworkers [36]. Speradine B and D from Ma et al. [39] actually correspond with speradine F and C, respectively, as described by Hu et al. [37]. Speradine C reported in Ma et al. [39] was actually a new compound possessing an unprecedented 6/5/6/5/5/6 hexacyclic system with a unique 4-oxo-1,3-oxazinane ring. In order to differentiate the metabolite previously isolated by Hu et al. [36], we decided to name this compound speradine I. Afterwards, five highly oxygenated CPA-related alkaloids, aspergillines A−E (Figure 1C), all having a rigid and sterically congested hexacyclic 6/5/6/5/5/5 indole-tetrahydrofuran-tetramate scaffold, were isolated from *Aspergillus versicolor* [40]. Aspergillines B and E possess a butanoic acid methyl ester moiety, whereas aspergilline C contains an extra isoprenoid moiety attached to the indole nucleus (Figure 1C). In addition, another group of CPA-related oxindoles, named cyclopiamides A−J, were isolated from a deep-sea-derived strain of *Penicillium commune* [41,42] (Figure 1D). Cyclopiamides H and I isolated in *P. commune* prove to be the same chemical entities with speradine B and aspergilline D, respectively. To avoid future confusion regarding the nomenclature of the CPA-related alkaloids, we suggest they are named as they were discovered chronologically (Figure 1; Table 1).

**Figure 2:**
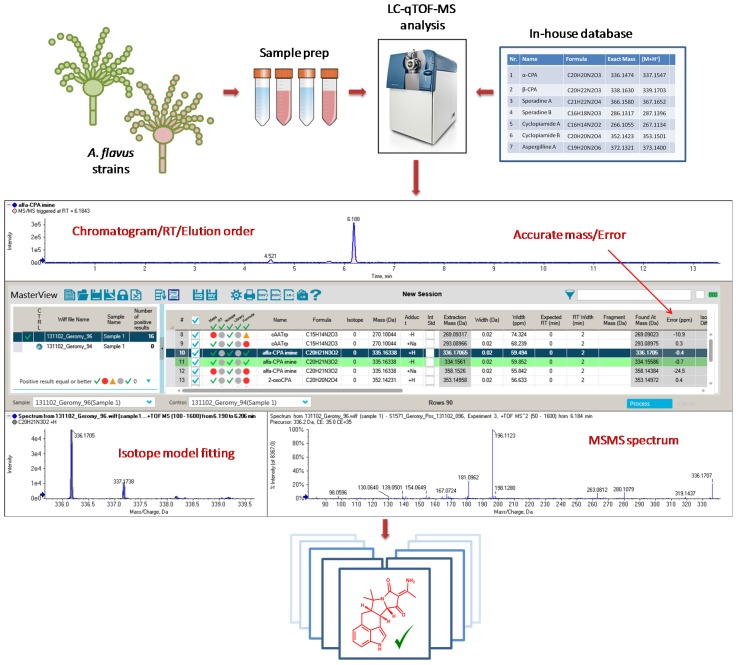
Dereplication workflow of already reported CPA-related alkaloids by using *MasterView^TM^ software* as an integrated package displaying: Extracted chromatogram (RT—retention time), mass accuracy (error—ppm), relative quantity (area—arbitrary units), isotope-model fitting and tandem mass spectrometry (MS/MS) spectra.

Since *Aspergillus flavus* is an important mycotoxigenic mold, and a very frequent food and feed contaminant with ubiquitous nature, the probability of human and animal exposure to CPA, as well as its associated health hazard, is higher compared to other fungal species. On the other hand, one method for preventing aflatoxin contamination of crops is by introducing a non-aflatoxigenic competitor strain of *A. flavus* to compete with natural aflatoxin-producing fungi. Although this approach may reduce aflatoxin levels in food and feed commodities, the accumulation of other mycotoxins such as CPA has been observed [43]. In this regard, it is of utmost importance to thoroughly investigate this fungus for its capability to produce known and yet unknown CPA-type alkaloids. This can be achieved through a dereplication strategy based on accurate mass high resolution mass spectrometry (HRMS) and fragmentation data [44]. Nowadays, accurate mass measurements, isotope-model fitting, tandem mass spectrometry (MS/MS) spectra and chemical databases are integrated in single software packages, thus allowing a fast and aggressive dereplication of known metabolites (Figure 2). A careful study of the fragmentation pattern of known compounds can be used to help detect and identify novel and previously unreported analogues. Hence, the main aim of this work was to investigate the diversity of the CPA family of alkaloids in different strains of *A. flavus* by accurate mass HRMS, thereby building knowledge towards a better assessment of the global *A. flavus* mycotoxin burden.

## 2. Results

### 2.1. Identification of Indole Cyclopiazonic Acid (CPA)-Type Derivatives

A dereplication approach based on accurate mass HRMS data, combined with a careful examination of fragmentation spectra, was applied to ascertain the presence of previously identified CPA-type alkaloids and to establish an unambiguous identification strategy for further screening work. The employed analytical methodology involves an untargeted data acquisition (consisting of full scan time-of-flight (TOF) HRMS survey and information-dependent acquisition, (IDA) MS/MS scans) and the processing of data using both targeted and untargeted approaches. An α-CPA reference standard was available, and therefore, this compound was identified in the fungal extracts by comparison of retention time, accurate mass HRMS and HRMS/MS data with the reference standard. A careful investigation and interpretation of α-CPA fragmentation data was the basis for the identification of other CPA-type alkaloids as described below.

**Figure 3:**
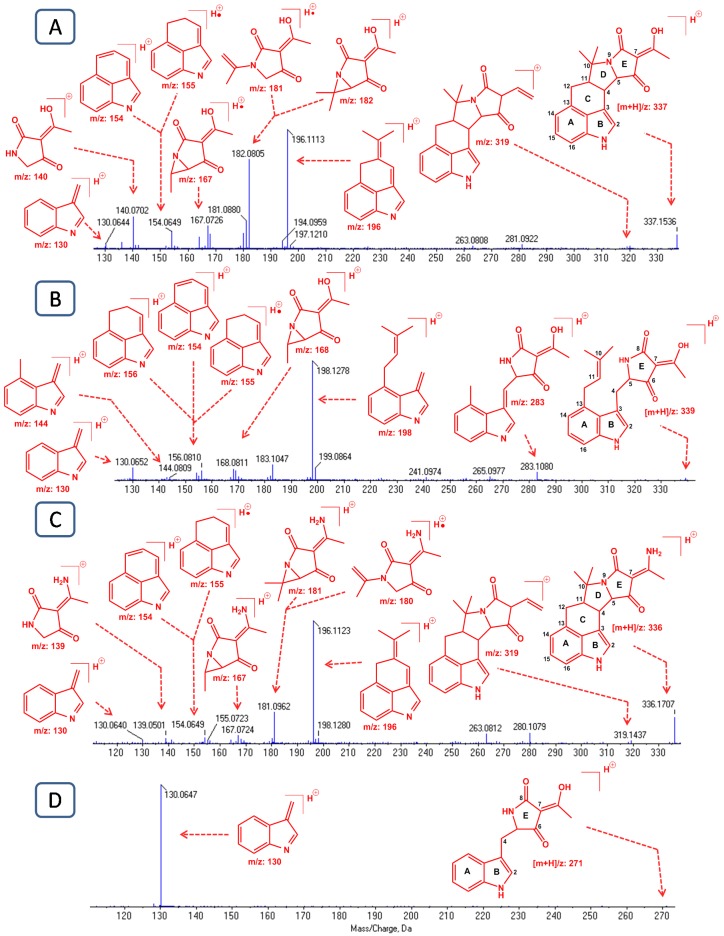
MS/MS spectra and putative structural fragments of: (**A**) α-CPA; (**B**) β-CPA; (**C**) α-CPA imine; (**D**) *cyclo*-acetoacetyl-l-tryptophan (cAATrp). The MS/MS spectra were acquired in IDA (information dependent acquisition) mode using a CE (collision energy) of 35 V with a collision energy spread (CES) of 15 V.

A perfect match was observed between the α-CPA MS/MS spectrum of the reference standard and that of the putative CPA in *A. flavus* extracts. A typical MS/MS spectrum of the protonated α-CPA ion ([*m* + H]^+^/*z* 337.1536; ∆ = −0.9 ppm) is shown in Figure 3A. The observed fragmentation pattern is also in accordance with reports from previous studies [1,45,46]. This spectrum, apart from the parent ion ([*m* + H]^+^/*z* 337), also shows some prominent fragment ions at *m*/*z* 196, 182, 167, 154 and 140 together with their “sibling species” at *m*/*z* 197, 181, 168, 155 and 141, thereby corroborating the findings from Holzapfel [1]. Generation of these metastable species most likely is achieved by ejection of a hydrogen atom from the main original fragment or vice versa [1]. For example, at least a part of the *m*/*z* 154 signal is yielded by proton ejection from *m*/*z* 155. High resolution mass measurements revealed that fragments at mass 154 and 155 correspond to the chemical formula of C_11_H_8_N^+^ and C_11_H_9_N^+^, respectively. Based on their chemical composition, these fragments must contain the indole system and three additional carbon atoms, resulting from the cleavage of the C4-C5 and C10-C11 bonds of ring D. The fragment ion at *m*/*z* 196 has the chemical composition of C_14_H_14_N^+^ and corresponds to the fragment ion 154 with three additional carbon atoms. This ion (*m*/*z* 196) arises by cleavage of the C4-C5 and C9-C10 bonds of ring D. Accurate mass measurements showed that the ion at mass 182 corresponds to the chemical formula C_9_H_12_NO_3_^+^. This fragment ion contains all the oxygen atoms of the parent ion, and therefore represents the tetramic acid moiety of the molecule as depicted in Figure 3A. The ion with *m*/*z* 167 is most likely formed by further ejection of a methyl group (-CH_3_) from the ion at *m*/*z* 182, whilst the mass 130 represents the indole nucleus of the molecule. On the other hand, the ion at *m*/*z* 140 represents ring E of α-CPA with the hydroxy-ethyl moiety (=C(OH)CH_3_) attached. Moreover, an ion with 18 Da difference (*m*/*z* 319) from the parent ion can be seen in the spectrum, which is attributed to the loss of a water molecule. β-CPA ([*m* + H]^+^/*z* 339.1700; ∆ = −0.9 ppm), also known as bissecodehydrocyclopiazonic acid, is a biosynthetic precursor of α-CPA with opened rings C and D. The MS/MS spectrum of this compound was similar to that previously reported by Holzapfel et al. [29], showing a peak at *m*/*z* 283 (C_16_H_15_N_2_O_3_^+^) which corresponds to a loss of the (CH_3_)C=CH_2_- group from the parent ion (Figure 3B). Another prominent peak, with *m*/*z* 198, can be seen in the spectrum, representing the indole nucleus together with the dimethylallyl moiety. This ion was generated by the cleavage of the C4-C5 bond. Moreover, accurate mass measurements of the product ion 198 revealed a chemical composition of C_14_H_16_N^+^, excluding oxygen functionalities which further supports its structural formula as depicted in Figure 3B. In the same spectrum, three other fragment ions can be seen at *m*/*z* 156 (C_11_H_10_N^+^), 155 (C_11_H_9_N^+^) and 154 (C_11_H_8_N^+^), which were also observed in the mass spectrum of α-CPA but with different abundances. The presence of these tricyclic ions in the fragmentation pattern of the seco-molecule β-CPA can be explained by the fact that initial fragments of the precursor ion subsequently undergo a cyclization step of the two side chains of the indole system. More precisely this cyclization process is carried out by formation of the C4-C11 bond in the fragment ion at *m*/*z*198 and closure of ring C (Figure S1). The ion at *m*/*z* 144 (C_10_H_10_N^+^), most likely arises from the ion of mass 198 by loss of the isoprenoid moiety ((CH_3_)C=CH_2_-).

α-CPA imine is a very similar chemical entity to α-CPA, in which the hydroxyl functionality is substituted with an amino group. The fact that these metabolites are closely related to each other is also demonstrated by their shared fragmentation behavior. The MS/MS spectrum of α-CPA imine ([*m* + H]^+^/*z* 336.1707; ∆ < 0.1 ppm) showed ions with *m*/*z* 319, 196, 154, 155 and 130 identical with those observed for α-CPA (Figure 3C). The fragment ions containing the amino group in their structure, namely *m*/*z* 181, 180 and 139, showed 1 Da difference with their corresponding fragments from the α-CPA molecule, which is consistent with the mass difference between -OH and -NH_2_ groups. The biosynthetic intermediate of α-CPA, *cyclo*-acetoacetyl-l-tryptophan (cAATrp), showed only one prominent fragment peak at *m*/*z* 130, corresponding to the indole nucleus of this metabolite (Figure 3D). Pseuboydone E was not detected in our samples.

### 2.2. Identification of Oxindole CPA-Type Derivatives

2-oxo-CPA is an oxygenated derivative of α-CPA. More precisely, it is an oxygen atom in the form of a keto-group that is added at the C2 position of the indole nucleus of the α-CPA. The MS/MS spectrum of 2-oxo-CPA ([*m* + H]^+^/*z* 353.1504; ∆ = 0.8 ppm) showed, besides the precursor ion at [*m* + H]^+^/*z* 353, characteristic fragments at *m*/*z* 335, 212, 182, 170, 154, 146 and 140 (Figure S2A). Since 2-oxo-CPA has an extra oxygen atom in its structure compared to its precursor, α-CPA, there is a mass difference of 16 Da between the two compounds. In this regard, all fragment ions in which the oxygenated indole system is incorporated show this 16 Da difference with their corresponding α-CPA fragments. Hence, fragment ions at *m*/*z* 335, 212, 170 and 146 are the oxygenated analogues of the fragment ions at *m*/*z* 319, 196, 154 and 130 in the MS/MS spectrum of α-CPA. On the other hand, peaks at *m*/*z* 182 and 140, which contain the tetramate moiety are identical to their corresponding fragments in the α-CPA MS/MS spectrum. The presence of the tricyclic fragment ion at *m*/*z* 154 may be justified by a possible cleavage of the hydroxyl group at C2 position of the ion at *m*/*z* 170.

**Figure 4:**
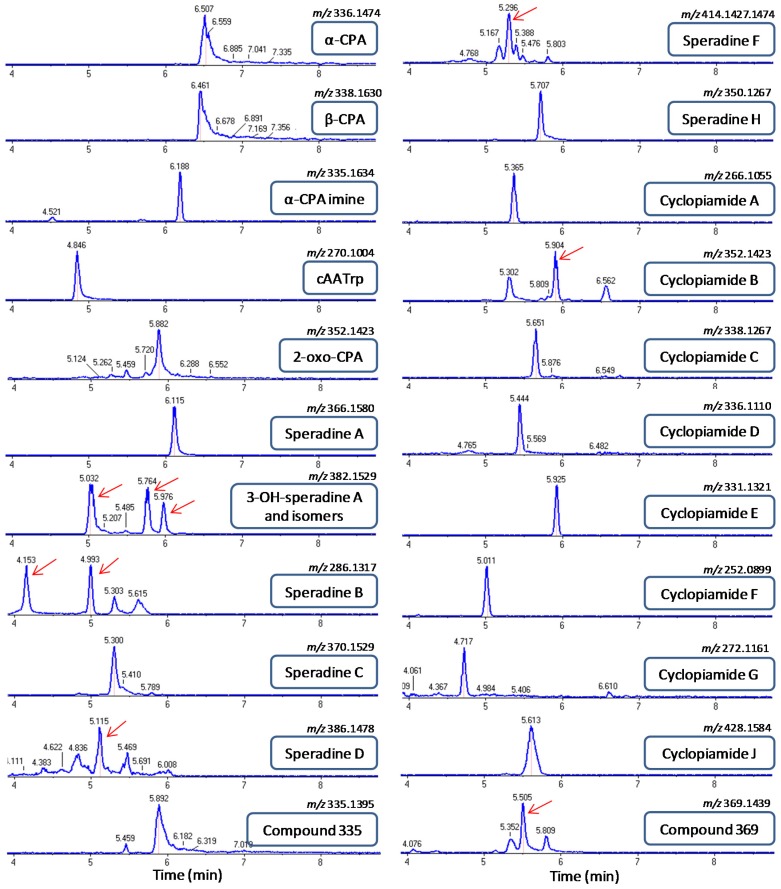
Extracted ion chromatograms (XIC) and elution order of CPA-type alkaloids detected in fungal extracts of different strains of *Aspergillus flavus*. The extraction mass window was set at 25 mDa. Red arrows indicate the correct retention time.

Speradine A also known as 1-*N*-methyl-2-oxo-CPA, is a methylated derivative of 2-oxo-CPA first reported in a marine-derived isolate of *A. tamarii*. The fragmentation pattern of speradine A ([*m* + H]^+^/*z* 367.1648; ∆ = −1.0 ppm) was similar to that of 2-oxo-CPA and α-CPA (Figure S2B). Due to the extra *N*-methyl group attached in the indole nucleus, speradine A has a 14 Da mass difference with 2-oxo-CPA. This 14 Da mass difference can be observed in all the typical fragments of speradine A (*m*/*z* 349, 325, 226 and 160) as compared to the MS/MS spectrum of 2-oxo-CPA. It is worth noting that HRMS and MS/MS data observed in our study for speradine A are in accordance with previous reports [33,34]. 3-Hydroxy-speradine A ([*m* + H]^+^/*z* 383.1610; ∆ = 2.0 ppm) is a hydroxylated derivative of speradine A, in which a hydroxyl functionality is added in the C3 position of the speradine A. The extraction of the mass corresponding to 3-hydroxy-speradine A, i.e., [*m* + H]/*z* 383.1610 resulted in three different peaks at RT 5.03, 5.76 and 5.97 min (Figure 4). The fragmentation pattern of the peaks at RT 5.76 and 5.97 min corresponded to the 3-OH-speradine A, whereas the metabolite eluting at RT 5.03 min had a slightly different fragmentation pattern, suggesting that the hydroxyl group may be attached at a different position. MS/MS spectrum of 3-OH-speradine A is depicted in Figure S2C. The two peaks with the mass of precursor ion and fragmentation pattern corresponding to 3-OH-speradine A can be explained by existence of different diastereoisomers.

Speradine B ([*m* + H]/*z* 287.1391; ∆ = −0.4 ppm) eluted at two different retention times, 4.15 and 4.99 min, both displaying identical MS/MS spectra with a typical fragmentation pattern for cyclopiazonic acid derivatives missing ring E (Figure S3A; Figure 4). The elution of speradine B at two different peaks can be justified by the existence of the same chemical compound in several stereoisomeric forms.

Speradine C has a very similar core structure with speradine B, except that in the N-6 position of speradine C a diketide moiety is attached. Based on this structural analogy, speradine C ([*m* + H]^+^/*z* 371.1600; ∆ = −0.2 ppm) underwent a very similar mode of fragmentation with that of speradine B, as depicted in Figure S3B. MS/MS spectrum of speradine C showed a peak at *m*/*z* 353, which corresponds to the loss of a water molecule (18 Da) from the parent ion. The fragment ion at *m*/*z* 287 represents the molecular ion of speradine B, which is generated after the loss of the diketide moiety from the N-6 position of speradine C. The rest of the fragments’ ions (i.e., *m*/*z* 226, 184, 169, 156 and 129) are identical with those in the fragmentation pattern of speradine B.

Speradine D is a closely related metabolite to speradine C showing a typical pathway of fragmentation for cyclopiazonic acids missing ring E and possessing a saturated ring C (Figure S3C). In the same fashion as speradine C, initially the OH group speradine D ([*m* + H]/*z* 387.1554; ∆ = 1.0 ppm) in the C3 position was lost through the elimination of a water molecule (−18 Da); subsequently, a cleavage of the N6-linked side chain occurred. The downstream fragmentation pathway of speradine D was identical with that of speradine B and C.

Speradine F is a highly oxygenated hexacyclic oxindole-tetrahidrofuran-tetramate metabolite having a very similar structural scaffold as the aspergillines isolated in fungal extracts of *A. versicolor*. Since speradine F is an *N*-methyl-2-oxo-indole with a saturated ring C, in the MS/MS spectrum of this compound ([*m* + H]^+^/*z* 415.1497; ∆ = −1.9 ppm), we could see the typical mass fragments for this subgroup of oxindoles like *m*/*z* 269, 226, 184 and 169, as were observed in the fragmentation pattern of speradine B, C and D (Figure S3D). Besides these typical ions, two other ions could be assigned in this MS/MS spectrum, namely the fragment ions at *m*/*z* 397 and 379. The fragment ion at *m*/*z* 397 is attributed to the disruption of ring F (tetrahydrofuran ring) as a water molecule, whilst the 379 ion is generated from the subsequent loss of a water molecule from the C7-linked side chain. On the other hand, speradine H ([*m* + H]^+^/*z* 351.1341; ∆ = −1.1 ppm) is a tetracyclic *N*-methyl-2-oxo-indole with an unsaturated ring C, thus exerting some typical fragments for this subgroup of oxindoles such as, *m*/*z* 267, 250, 222, 207 and 194 (Figure S3E).

Cyclopiamide A ([*m* + H]^+^/*z* 267.1132; ∆ = −0.7 ppm) and cyclopiamide B ([*m* + H]^+^/*z* 353.1498; ∆ = −0.8 ppm), both tetracyclic *N*-methyl-2-oxo-indoles with an unsaturated ring C, shared the same fragmentation pathway as speradine H (Figure S4A,B). Cyclopiamide C ([*m* + H]^+^/*z* 339.1339; ∆ = −0.2 ppm), cyclopiamide D ([*m* + H]^+^/*z* 337.1178; ∆ = −2.9 ppm) and cyclopiamide F ([*m* + H]^+^/*z* 253.0966; ∆ = −3.0 ppm), all belonging to the subgroup of 2-oxo-indoles with an unsaturated ring C, shared the same fragmentation pathway, with the most prominent fragments at *m*/*z* 253, 236, 208, 180 and 165 (Figures S4C,D and S5B). Cyclopiamide E is actually a pentacyclic *N*-methyl-2-oxo-indole with a very characteristic ring E, a 4-oxo-1,3-diazine, which seems to influence the fragmentation pattern of the parent ion. Hence, although it belongs to *N*-methyl-2-oxo-indoles with unsaturated ring C, cyclopiamide E ([*m* + H]^+^/*z* 332.1409; ∆ = 2.7 ppm) showed a unique pattern of fragmentation with the most prominent fragment peaks at *m*/*z* 317, 288, 274 and 249 (Figure S5A).

Cyclopiamide G is a 2-oxo-indole with a saturated ring C, thus sharing some key structural fragments (*m*/*z* 212 and 170) with 2-oxo-CPA. Furthermore, in the MS/MS spectrum of cyclopiamide G ([*m* + H]^+^/*z* 273.1233; ∆ = −1.5 ppm), an ion with *m*/*z* 255 demonstrates the loss of a hydroxyl group from the C3 position of the parent molecule (Figure S5C). Cyclopiamide J ([*m* + H]^+^/*z* 429.1656; ∆ = −0.9 ppm) underwent a similar fashion of fragmentation as speradine F, because they share almost the same chemical scaffold with a small difference in the C7-linked side chain. Hence, the typical fragments at *m*/*z* 397, 379, 269, 226, 184 and 169 can be observed in the MS/MS spectrum of cyclopiamide J (Figure S5D). None of the aspergillines could be detected in the different strains of *A. flavus*.

### 2.3. Identification of Previously Unreported CPA-Type Derivatives

To investigate other derivatives that were not included in our list of target CPA-type alkaloids, the MS/MS data were checked for the presence of diagnostic ions of this class of compounds (Table S2). Besides the above known compounds, this untargeted analysis uncovered two other metabolites, *m*/*z 335.1395* and *m*/*z 369.1439*, which shared the same fragmentation pattern and whose MS/MS spectra encompassed diagnostic ions of the CPA-type alkaloids. Accurate mass measurements revealed that the metabolite at [*m* + H]^+^/*z* 335.1395 (∆ = 1.4 ppm) corresponds to the chemical formula C_20_H_19_N_2_O_3_, possessing two protons less than the original molecule of α-CPA. These two protons’ (2 Da) difference implies an extra double bond in the chemical scaffold of α-CPA. The location of this double bond is most likely in ring C of the molecule because fragment ions at *m*/*z* 130 (ring A and B), *m*/*z* 168 and 182 (ring D and E) are the same as described in the MS/MS spectrum of α-CPA, whilst the fragments at *m*/*z* 194, 223, 251, 317 containing ring C in their structure exhibit that 2 Da difference with their corresponding fragments from the α-CPA MS/MS spectrum (Figure S6A). On the other hand, the metabolite eluting at RT 5.5 min had the chemical formula C_20_H_21_N_2_O_5_ ([*m* + H]^+^/*z* 369.1439; ∆ = −1.6 ppm). Chemical composition as well as fragmentation pattern of this metabolite provided enough evidence to be assigned as a C3-hydroxylated analogue of 2-oxo CPA (Figure S6B).

### 2.4. Screening of CPA-Type Alkaloids in Different *A. flavus* Strains

Based on the data described above, a screening of CPA-type alkaloids was performed on a set of 55 *A. flavus* strains. In total, 22 CPA-type alkaloids were identified in extracts of the strains investigated, demonstrating the great potential of this ubiquitous fungus in producing secondary metabolites (Table 2). Of these metabolites, 13 have been previously reported in other fungi, but here they are reported for the first time in *A. flavus*. We also report the occurrence of two novel CPA-related metabolites in these samples.

## 3. Discussion

Chemically, α-CPA is a hybrid prenylated indole alkaloid, structurally characterized by a rigid pentacyclic (6/5/6/5/5) skeleton bearing a unique heterocyclic pyrrolidine-2,4-dione (tetramic acid) motif as the main part of its pharmacophore. This mycotoxin does not contain any *O*-methyl or *N*-methyl groups in its structural scaffold and is characterized by the ability to form an intramolecular hydrogen bond due to its keto-enol tautomerism. Since α-CPA is a very well-known mycotoxin, its identification in *A. flavus* fungal extracts was straightforward, and was also supported by data from previous work [1,3,45,46]. The same mode of fragmentation was observed in the MS/MS spectrum of α-CPA and those of other indole derivatives (β-CPA, α-CPA imine and cAATrp) (Figure 3). The identification of these compounds was also straightforward, as they share the same scaffold with α-CPA. On the other hand, the identification of the oxindole CPA-type alkaloids was more complex due to their high chemical diversity in terms of different structural scaffolds. Moreover, this subclass of alkaloids is less studied compared to α-CPA itself and other indole derivatives. For this reason, and also to simplify the identification, we subdivided the oxindole subclass of CPA-type alkaloids in four different chemical groups: (i) 2-oxindoles with saturated ring C (2-oxo-CPA and cyclopiamide G); (ii) 2-oxindoles with unsaturated ring C (cyclopiamide C, cyclopiamide D and cyclopiamide F); (iii) *N*-methyl-2-oxindoles with saturated ring C (speradine A, 3-hydroxy-speradine A, speradines B-D, speradine F, cyclopiamide H and cyclopiamide J); and (iv) *N*-methyl-2-oxindoles with unsaturated ring C (speradine E, speradine H, cyclopiamide A, cyclopiamide B, and cyclopiamide E). The MS/MS spectra demonstrated that all the metabolites belonging to the same chemical group share two or three typical fragments that reflect the core structure of the respective chemical group (Table S2), and which can therefore be used as diagnostic ions. This chemical classification and the diagnostic ion approach facilitated the dereplication of oxindoles included in our study, and it is also a useful methodology for the identification of yet unknown CPA-type oxindoles. Besides accurate mass measurements and MS/MS data, other analytical parameters like elution order and isotope-model fitting prove to be complementary identification aids. For instance, in our study, speradine A eluted between α-CPA and 2-oxo-CPA, which actually represents the same elution pattern as reported by other researchers [33]. Similar successful dereplication approaches based on TOF technologies have been reported previously [44,47,48].

Production of polyketide-amino acid hybrid metabolites, as is the case for α-CPA, is catalyzed by PKS-NRPS enzymes. PKS-NRPSs are complex and multi-domain enzymes characterized as having the ability to synthesize highly diverse chemical groups of secondary metabolites [49]. The genome of *A. flavus* is predicted to harbor two PKS-NRPS hybrid gene clusters. One (cluster #55) is the gene cluster of CPA [9], while the other one (cluster #23) has been shown to be responsible for the production of a series of 2-pyridones [50,51]. As indicated above, the CPA gene cluster in *A. flavus* NRRL 3357 contains only three functional genes and a Zn_2_Cys_6_ transcription factor-encoding gene (*ctfR1*), which seems to be inactive [10]. On the other hand, our study demonstrated a huge chemical diversity within the CPA family of mycotoxins. Therefore, different *A. flavus* strains have the ability to produce many more metabolites apart from α-CPA and its precursors (Table 2). Although it is hard to predict if all the identified metabolites are linked with the CPA gene cluster, the chemical scaffold resemblance strongly suggests a convergent biochemical origin. It is likely that the genetic material reported for the CPA gene cluster in NRRL 3357 is not sufficient to explain the chemical diversity found in other strains of *A. flavus*. Hence, most likely other *A. flavus* strains like S295, S283, S1637, and S1544 possess additional genes within their CPA clusters that may catalyze extra biochemical steps in the biosynthetic pathway of CPA. This concept was already demonstrated in *A. oryzae* NBRC 4177 in the case of 2-oxoCPA. A cytochrome P450 oxidase gene (*cpaH*) located within a CPA cluster in NBRC 4177 was shown to mediate the conversion of α-CPA to 2-oxoCPA [32]. In the same fashion, it was reported that a strain of *A. tamarii* (NBRC 4099) harbors the gene *cpaM*, which encodes for a *N*-methyltransferase involved in the synthesis of 1-*N*-methyl-2-oxoCPA (speradine A) from 2-oxoCPA [33]. The inability of speradine A production in *A. oryzae* is caused by mutations or partial deletions in *cpaM*. In analogy with aforementioned findings, we can speculate that the homolog genes of *cpaH* and *cpaM* may be also present in the CPA gene cluster of 2-oxo-CPA and speradine A-producing strains of *A. flavus*. In this regard, the CPA gene cluster seems more genetically diverse than expected, especially when we consider the identification of other complex CPA-type alkaloids like speradine F, cyclopiamide E, cyclopiamide J, the aspergillines, etc. Additional genetic data are needed to support the occurrence of these metabolites in *A. flavus* fungal extracts. Nevertheless, we cannot exclude the possibility of involvement in the CPA-biosynthetic network of enzymes that are encoded by genes located outside the CPA gene cluster. Moreover, there is also the possibility of non-enzymatic generation of metabolites under different culture conditions or during various sample treatment procedures.

The tetramic acid structural moiety is a very important nitrogen-containing heterocycle that is often the pharmacophore to interact with various biological targets, thus it exhibits a wide range of biological and toxicological activities [52,53,54]. Apart from α-CPA, 2-oxoCPA and speradine A, for which the SERCA-blocking activity was clearly demonstrated, the toxicological profile for the rest of the CPA-type metabolites is unknown [4,12,34]. In this context, it is important both to evaluate the actual toxicological potential of these CPA-related alkaloids and to get an idea about their contributions in the overall CPA toxicity. Moreover, there are no data about their occurrences in food and feed commodities, which further complicates our understanding about human and animal exposure to CPA contamination. Hence, conducting survey studies of CPA-type mycotoxins in different food and feed matrices might be necessary to decipher the real impact of CPA contamination in human and animal health, especially considering the fact that CPA is always overshadowed by concern about aflatoxin contamination.

One of the main approaches for pre-harvest control of aflatoxin contamination in crops is the introduction of non-aflatoxigenic *A. flavus* isolates into agricultural fields to displace aflatoxin-producing strains. The necessary features for a potential *A. flavus* strain to be used as a biocontrol agent include ability to grow rapidly, to be adapted for specific plant colonization, and to produce sclerotia for long-term survival in the fields [55,56]. From a toxicological point of view, the only seemingly important feature for a biocontrol strain is the inability to produce aflatoxins. However, the inability to produce other relevant mycotoxins, among which are α-CPA and our newly identified CPA analogues, is highly desirable due to the unknown long-term and cumulative toxicological effects of these metabolites. Thus, a careful screening of these metabolites is obviously very important in order to avoid any possible inadvertent mycotoxicoses from the introduction of biocontrol agents into agricultural fields. Also, increased efforts to assess the genetic stability of the *A. flavus* strains used in biocontrol products should be undertaken. This is highly relevant since there is now evidence for a sexual stage in several aflatoxigenic *Aspergillus* species [57,58]. Hence, the possibility of re-gaining aflatoxin- and/or CPA-producing properties through sexual recombination is a fact that should not be neglected. The same concern of exposure to CPA-type mycotoxins can be made regarding *A. oryzae* strains which are being used extensively in the food fermentation industry [59].

Data from the present work show a conspicuous presence of CPA and its derivatives in the different *A. flavus* strains investigated, and highlight the previously mentioned need for a thorough assessment of the actual impact of contamination of crops with CPA-type mycotoxins on human and animal health. Two commercially available biocontrol strains (AF36 and NRRL 21882) were included in this study. AF36 was originally isolated from a cotton field in Arizona and was approved for application on cotton in Arizona. Loss of aflatoxigenicity in AF36 is the result of a nonsense mutation in *pksA* (*aflC*), a pathway gene in aflatoxin biosynthesis [60]. AF36 has an otherwise full aflatoxin cluster. AF36 does have a fully functional CPA cluster, and although it is effective at excluding toxigenic strains and reducing AF levels, this strain is reported to significantly increase CPA accumulation in food and feed commodities [61]. This is consistent with our findings which demonstrated the ability of AF36 to produce α-CPA and a series of other CPA-like alkaloids, including its precursors (cAATrp and β-CPA), 2-oxoCPA, cyclopiamide C, F, G and the two other previously unreported derivatives (see 2.3). Remarkably, our data uncovered the presence of the CPA family of mycotoxins in fungal extracts of NRRL 21882, which is reported to lack both the aflatoxin and CPA gene clusters [61]. These findings could support the phenomenon of heterokaryosis and vertical transmission of cryptic alleles in *A. flavus* in NRRL 21882. Olarte et al. [62] reported that it is possible for some *A. flavus* to possess cryptic alleles, particularly when typically masked genes are amplified because the dominant genome lacks the gene(s) of interest. Moore et al. [63] were able to amplify and sequence a portion of the *aflW*/*aflX* region in NRRL 21882. This indicates NRRL 21882 may be heterokaryotic with a low-copy genome possessing cryptic alleles for a functional CPA gene cluster, thereby making this particular strain a CPA producer even though it is missing the entire subtelomeric region of chromosome 3. Existence of similar non-parental cryptic alleles has been reported also in other fungal species [64,65].

It is worth noting that besides their toxicological relevance, there is currently a revival of interest for pharmacological application of CPA-type pharmacophores. As stated above, α-CPA is a specific nanomolar inhibitor of mammalian SERCA-1. This SERCA-inhibiting activity of α-CPA is being exploited extensively for various pharmacological purposes. Thus, Kotsubei et al. [66], by using α-CPA as an experimental inhibitor, demonstrated sufficient differences in active binding sites between mammalian and bacterial SERCA. These differences in the CPA pocket between mammalian and bacterial Ca^2+^-ATPase suggest that a bacterial-specific CPA derivative could be developed, hence making the bacterial SERCA a potential drug target. Moreover, α-CPA has been shown to possess clear antiviral activity as well as causing perturbations in cardiac ventricular myocytes [67,68]. Based on these concepts, CPA-type alkaloids identified in this study, and/or other derivatives which may be discovered or synthesized in the future, could be useful bioactive scaffolds to be employed in future pharmacological applications.

## 4. Conclusions

Accurate mass high resolution mass spectrometry (HRMS), combined with a careful investigation of fragmentation patterns, proved to be a suitable dereplication strategy for the identification of cyclopiazonic acid (CPA)-type alkaloids. This approach resulted in our finding 22 CPA-type alkaloids in *A. flavus* cultures. Two of these metabolites are new discoveries as they have never been reported. Though the other 20 compounds have been previously identified in different fungi, 13 of these metabolites were identified for the first time in *A. flavus*. These results provide a better insight into the diversity of CPA-type alkaloids in *A. flavus* and raise concerns about the extent of the overall *A. flavus* mycotoxin problem. The described identification strategy can be applied in programs aiming to assess the occurrence of this type of mycotoxin in food and feed commodities. Our results also demonstrate the great potential of *A. flavus* in producing a myriad of secondary metabolites, highlighting the need for a more thorough investigation into potential mycotoxicity of non-aflatoxigenic strains that are currently being used in biocontrol strategies.

## 5. Materials and Methods

### 5.1. Chemicals and Materials

Methanol (MeOH) and acetonitrile (ACN), LC-MS grade, were obtained from Biosolve (Valkenswaard, the Netherlands), whereas HPLC-grade MeOH was from VWR International (Zaventem, Belgium). Ethyl acetate (EtOAc), dichloromethane (DCM) and acetone (dimethyl ketone (DMK)) were purchased from Acros Organics (Geel, Belgium). Sigma-Aldrich (Bornem, Belgium) supplied ammonium formate (HCOONH_4_). Formic acid (HCOOH, Merck, Darmstadt, Germany) was used. Ultrapure H_2_O was produced by a Milli-Q Gradient System (Millipore, Brussels, Belgium). Ultrafree^®^-MC centrifugal filter units (0.22 μm) from Millipore (Bedford, MA, USA) were used. Sigma-Aldrich supplied agar, corn steep solids, dextrose, peptone, sucrose, yeast extract, dipotassium hydrogen phosphate trihydrate (K_2_HPO_4_∙3H_2_O), magnesium sulfate heptahydrate (MgSO_4_∙7H_2_O), and iron(II) sulfate heptahydrate (FeSO_4_∙7H_2_O). Triton X-100, potassium chloride (KCl), and sodium nitrate (NaNO_3_) were from Merck.

### 5.2. Strains and Growth Conditions

*A. flavus* strains used in this study are listed in Table S1 as supplementary data. Conidia of each respective strain were inoculated on solid Wickersham media (≈25 mL of medium per plate, *D* = 10 cm) which contains 2.0 g yeast extract, 3.0 g peptone, 5.0 g corn steep solids, 2.0 g dextrose, 30.0 g sucrose, 2.0 g NaNO_3_, 1.0 g K_2_HPO_4_∙3H_2_O, 0.5 g MgSO_4_∙7H_2_O, 0.2 g KCl, 0.1 g FeSO_4_∙7H_2_O, 15.0 g agar per litre (pH 5.5). All cultures were incubated at 28 °C in the dark for 7 days.

### 5.3. Sample Preparation

The fungal colonies and agar were cut into small pieces with a scalpel and these were subsequently transferred to a 500 mL screw-cap Ehrlenmeyer flask. Metabolites were extracted with 30 mL MeOH:DCM:EtOAc 10:20:30, (*v*/*v*/*v*). The samples were agitated for 60 min on an Agitelec overhead shaker (J. Toulemonde and Cie, Paris, France). A total of 4 mL of extract was transferred to a glass tube and evaporated under a stream of nitrogen. The residue was reconstituted with 200 μL MeOH:ACN:H_2_O 30:30:40, (*v*/*v*/*v*), and centrifuged in an Ultrafree^®^-MC centrifugal device for 5 min at 14,000× *g*.

### 5.4. UHPLC-qTOF-MS Analysis

The experiments were carried out using a hybrid Q-TOF MS instrument, the AB SCIEXTripleTOF^®^4600 (AB Sciex, Concord, ON, Canada), equipped with a DuoSpray^TM^ and coupled to an Eksigent ekspert™ ultraLC 100-XL system. The DuoSpray^TM^ ion source (consisting of both electrospray ionization (ESI) and atmospheric pressure chemical ionization (APCI) probes) was operated in the positive ESI mode (ESI^+^). The APCI probe was used for automated mass calibration using the Calibrant Delivery System (CDS). The CDS injects a calibration solution matching the polarity of ionization, and calibrates the mass axis of the TripleTOF^®^ system in all scan functions used (MS and/or MS/MS). The Q-TOF HRMS method consisted of a full scan TOF survey (dwell time 100 ms, 100–1600 Da) and a maximum number of eight IDA MS/MS scans (dwell time 50 ms). The MS parameters were as follows: curtain gas (CUR) 25 psi, nebulizer gas (GS 1) 50 psi, heated gas (GS 2) 60 psi, ion spray voltage (ISVF) 5.5 kV, interface heater temperature (TEM) 500 °C, Collision Energy (CE) 10 V and declustering potential (DP) 70 V. For the IDA MS/MS experiments, a CE of 35 V was applied with a collision energy spread (CES) of 15 V. An Eksigentekspert™ ultraLC 100-XL system was used for separation. The column was a ZORBAX RRHD Eclipse Plus C18 (1.8 μm, 2.1 × 100 mm) from Agilent Technologies (Diegem, Belgium). The mobile phase consisted of H_2_O:MeOH (95:5, *v*/*v*) containing 0.1% HCOOH and 10 mM HCOONH_4_ (solvent A) and MeOH:H_2_O (95:5, *v*/*v*) containing 0.1% HCOOH and 10 mM HCOONH_4_ (solvent B). The gradient elution program for LC-qTOF HRMS analyses was applied as follows: 0–0.5 min: 0% B, 0.5–7 min: 0%–99% B, 7–9 min: 99% B, 9–10 min: 99%–0% B, 10–14 min: 0% B. The flow rate was 0.4 mL/min. The column temperature was set at 40 °C and temperature of the autosampler was 4 °C. 5 μL of sample were injected. The instrument was controlled by Analyst^®^ TF 1.6 software, while data processing was carried out using PeakView^®^ software version 2.0 and MasterView^TM^ software version 1.0 (all from AB Sciex).

## Figures and Tables

**Table 1:** CPA-type alkaloids identified in different fungal sources.

Compound	Name	Formula	Exact Mass	Source	Reference
**1**	α-CPA	C_20_H_20_N_2_O_3_	336.1474	Various *Aspergillus* and *Penicillium* species	[1,3,29]
**2**	Iso-α-CPA	C_20_H_20_N_2_O_3_	336.1474	*Aspergillus flavus*	[30]
**3**	β-CPA	C_20_H_22_N_2_O_3_	338.1630	Various *Aspergillus* and *Penicillium* species	[8,9,29]
**4**	cAATrp	C_15_H_14_N_2_O_3_	270.1004	Various *Aspergillus* and *Penicillium* species	[8,9,10,11]
**5**	α-CPA imine	C_20_H_21_N_3_O_2_	335.1634	*Penicillium cyclopium*	[29]
**6**	Pseuboydone E	C_19_H2_0_N_2_O_3_	324.1474	*Pseudallescheria boydii*	[31]
**7**	2-oxoCPA	C_20_H_20_N_2_O_4_	352.1423	*Aspergillus oryzae*	[32,33]
**8**	Speradine A	C_21_H_22_N_2_O_4_	366.1580	*A. tamarii*, *A. oryzae*	[33,34]
**9**	3-OH-speradine A	C_21_H_22_N_2_O_5_	382.1529	*A. tamarii*	[35]
**10**	Speradine B	C_16_H_18_N_2_O_3_	286.1317	*A. oryzae*, *A. flavus*	[36,39]
**11**	Speradine C	C_20_H_22_N_2_O_5_	370.1529	*A. oryzae*, *A. flavus*	[36,39]
**12**	Speradine D	C_20_H_22_N_2_O_6_	386.1478	*A. oryzae*	[36]
**13**	Speradine E	C_20_H_18_N_2_O_5_	366.1216	*A. oryzae*	[36]
**14**	Speradine F (Pencamedine A)	C_21_H_22_N_2_O_7_	414.1427	*A. oryzae*, *P. commune*, *A. flavus*, *P. camemberti*	[37,38,39,41]
**15**	Speradine H	C_20_H_18_N_2_O_4_	350.1267	*A. oryzae*, *P. commune*	[37,41]
**16**	Speradine I	C_21_H_22_N_2_O_7_	414.1427	*A. flavus*	[39]
**17**	Aspergilline A	C_19_H_20_N_2_O_6_	372.1321	*A. versicolor*	[40]
**18**	Aspergilline B	C_23_H_26_N_2_O_8_	458.1689	*A. versicolor*	[40]
**19**	Aspergilline C	C_24_H_28_N_2_O_6_	440.1947	*A. versicolor*	[40]
**20**	Aspergilline D (Cyclopiamide I)	C_21_H_24_N_2_O_7_	416.1584	*A. versicolor*, *P. commune*	[41,42]
**21**	Aspergilline E	C_25_H_30_N_2_O_9_	502.1951	*A. versicolor*	[40]
**22**	Cyclopiamide A	C_16_H_14_N_2_O_2_	266.1055	*P. cyclopium*, *P. commune*, *A. flavus*	[39,41,42]
**23**	Cyclopiamide B	C_20_H_20_N_2_O_4_	352.1423	*P. commune*	[41]
**24**	Cyclopiamide C	C_19_H_18_N_2_O_4_	338.1267	*P. commune*	[41]
**25**	Cyclopiamide D	C_19_H_16_N_2_O_4_	336.1110	*P. commune*	[41]
**26**	Cyclopiamide E	C_20_H_17_N_3_O_2_	331.1321	*P. commune*	[41]
**27**	Cyclopiamide F	C_15_H_12_N_2_O_2_	252.0899	*P. commune*	[41]
**28**	Cyclopiamide G	C_15_H_16_N_2_O_3_	272.1161	*P. commune*	[41]
**29**	Cyclopiamide H	C_16_H_18_N_2_O_3_	286.1317	*A. oryzae*, *P. commune*, *A. flavus*	[36,39,41]
**30**	Cyclopiamide J	C_22_H_24_N_2_O_7_	428.1584	*P. commune*	[41]

**Table 2:** CPA-type alkaloids detected in our study.

*A. flavus* Strains (SRRC)	CPA-Type Alkaloids																					
	α-CPA	β-CPA	α-CPA Imine	cAATrp	2-oxoCPA	Speradine A	3-OH-Speradine A	Speradine B	Speradine C	Speradine D	Speradine F	Speradine H	Cyclopiamide A	Cyclopiamide B	Cyclopiamide C	Cyclopiamide D	Cyclopiamide E	Cyclopiamide F	Cyclopiamide G	Cyclopiamide J	Compound 335	Compound 369
0038	++	++	++	+	++	+	-	-	-	-	++	-	-	-	++	+	-	+	+	-	++	++
0141	+	-	-	-	+	++	+	-	+	-	-	-	-	+	-	-	-	-	-	-	-	-
0144	+++	++	-	++	++	+	+	-	-	-	-	-	-	++	++	+	-	+	+	-	++	++
0150	+++	++	++	++	++	+	+	-	-	-	-	-	-	-	++	-	-	++	+	-	++	++
0151	-	-	-	-	-	-	-	-	-	-	-	-	-	-	-	-	-	-	-	-	-	-
0167	+++	++	+	++	++	-	-	-	-	-	-	-	-	-	-	-	-	-	-	-	++	++
0283	++	++	-	++	++	++	++	++	++	+	++	-	++	++	++	+	-	+	+	++	++	++
0295	++	++	-	++	++	++	+++	++	++	+	++	++	++	++	+	+	++	+	+	++	++	++
1000F	+++	++	-	++	++	-	-	-	-	-	-	-	-	-	-	-	-	+	-	-	++	++
1006	++	++	++	++	+	++	++	-	-	-	-	-	-	+	+	+	-	+	-	-	++	++
1020	+++	++	++	++	+	-	-	-	-	-	-	-	-	-	++	-	-	++	+	-	++	++
1021	++	++	+	+	++	-	-	-	-	-	-	-	-	-	-	-	-	-	-	-	-	++
1055	+++	+	+	++	++	-	-	-	-	-	-	-	-	-	++	++	-	++	+	-	++	++
1071	++	++	-	-	+	-	-	-	-	-	-	-	-	-	+	-	-	+	-	-	+	+
1098	++	+	-	++	-	-	-	-	-	-	-	-	-	-	+	-	-	++	-	-	++	+
1118	-	-	-	-	-	-	-	-	-	-	-	-	-	-	-	-	-	-	-	-	-	-
1187	++	++	-	++	++	-	-	-	-	-	-	-	-	-	++	-	-	-	-	-	++	++
1299	++	++	++	++	++	-	-	-	-	-	-	-	-	-	+	-	-	+	+	-	++	++
1356	++	-	++	++	++	++	-	-	-	-	-	-	-	-	+	-	-	++	+	-	++	++
1357	++	++	-	++	+	++	++	++	-	-	-	++	++	-	++	-	-	+	-	++	+	-
1533	++	++	-	++	++	-	-	-	-	-	-	-	-	-	++	-	-	+	+	-	++	++
1534	++	++	-	+	-	-	-	-	-	-	-	-	-	-	-	-	-	+	-	-	-	-
1540	++	+	-	+	++	++	+	-	-	-	-	-	-	-	-	-	-	-	-	-	+	++
1541	++	-	-	++	-	-	-	-	-	-	-	-	-	-	++	-	-	-	-	-	-	-
1543	++	-	-	++	-	++	++	+	+	-	-	-	-	+	-	-	-	+	-	-	-	+
1544	++	++	-	++	++	++	++	++	++	++	++	++	++	++	+	-	++	++	-	++	++	++
1545	+++	++	++	++	++	-	-	-	-	-	-	-	-	-	++	+	-	++	+	-	++	++
1547	++	++	-	++	-	++	++	++	++	-	++	-	++	++	-	-	-	-	-	++	++	-
1552	++	-	-	-	+	++	++	-	+	-	-	-	-	-	-	-	-	-	-	-	-	+
1553	++	+	+	+	++	-	-	-	-	-	++	-	-	-	++	-	-	++	+	-	++	++
1554	+++	+	+	++	++	+	-	-	-	-	-	-	-	-	++	-	-	-	-	-	++	++
1557	++	++	-	++	+	++	++	++	++	-	++	++	++	++	-	-	++	-	-	++	++	-
1558	++	++	-	++	++	-	-	+	-	-	-	-	-	-	++	+	-	+	+	-	++	++
1559	+++	++	+	++	++	-	-	-	-	-	-	-	-	-	++	-	-	++	+	-	++	++
1565	++	++	-	+	+	-	-	-	-	-	-	-	-	-	+	+	-	-	-	-	++	++
1566	+++	++	-	+	++	-	-	-	-	-	-	-	-	-	-	-	-	-	-	-	++	++
1568	-	-	-	-	-	-	-	-	-	-	-	-	-	-	-	-	-	-	-	-	-	-
1571	++	++	++	++	+	++	+++	++	++	+	+	-	++	++	+	-	+	+	-	++	+	++
1573	+++	++	-	+	+	-	-	-	-	-	-	-	-	-	++	+	-	+	+	-	++	++
1574	+++	++	++	+	++	-	-	-	-	-	-	-	-	-	+	+	-	++	+	-	++	++
1575	++	++	-	++	-	++	++	++	++	-	++	++	++	++	-	-	++	-	-	++	++	-
1576	++	++	+	++	++	++	++	-	+	-	-	-	-	-	+	+	-	+	+	-	++	++
1578	+++	++	++	++	++	-	-	-	-	-	-	-	+	-	++	+	-	+	-	-	++	++
1591	++	++	-	++	++	++	+++	++	++	-	++	++	++	++	+	+	+	+	-	++	++	++
1626	++	++	+	++	++	++	++	-	+	-	-	-	-	-	++	+	-	+	+	-	++	++
1637	++	++	+	++	++	++	++	++	++	+	++	-	++	++	++	-	++	+	+	+	+	++
2000	++	-	-	++	++	-	-	-	-	-	-	-	-	-	++	-	-	-	-	-	+	++
2001	++	++	+	+	+	-	-	-	-	-	-	-	-	-	++	-	-	++	-	-	+	++
2033	+++	++	++	+	++	-	-	-	-	-	-	-	-	-	++	+	-	+	+	-	++	++
2035	+++	++	-	-	++	-	-	-	-	-	-	-	-	++	-	+	-	+	+	-	++	++
2114	-	-	-	-	-	-	-	-	-	-	-	-	-	-	-	-	-	-	-	-	-	-
2115	+++	++	++	++	++	-	-	-	-	-	-	-	-	-	-	-	-	+	+	-	++	-
2118	++	-	++	+	++	+	-	-	-	-	-	-	-	-	+	-	-	++	+	-	++	++
2524	+++	++	-	+	++	-	-	-	+	-	-	-	-	-	++	+	-	+	+	-	++	++
2711	+++	-	-	++	++	-	-	-	-	-	-	-	-	-	+	-	-	+	-	-	++	++

“+”, Peak area ≤ 10^4^; “++”, 10^4^ < Peak area < 10^6^; “+++”, Peak area ≥ 10^6^; “-“ not detected; SRRC-Southern Regional Research Center; CPA-cyclopiazonic acid; The presence of compounds was verified with MS/MS spectra as described under Section 2.1, Section 2.2 and Section 2.3.

## Supplementary Materials

The following are available online at www.mdpi.com/2072-6651/9/1/35/s1, Figure S1: Cyclization mechanism of the ions at *m/z* 156, 155 and 154 in the fragmentation pathway of β-CPA, Figure S2: MS/MS spectra and structural fragments of: A. 2-oxo-CPA; B. speradine A, and C. 3-hydroxy-speradine A, Figure S3: MS/MS spectra and structural fragments of: A. speradine B; B. speradine C; C. speradine D; D. speradine F, and E. speradine H, Figure S4: MS/MS spectra and structural fragments of: A. cyclopiamide A; B. cyclopiamide B; C. cyclopiamide C, and D. cyclopiamide D, Figure S5: MS/MS spectra and structural fragments of: A. cyclopiamide E; B. cyclopiamide F; C. cyclopiamide G, and D. cyclopiamide J, Figure S6: MS/MS spectra and structural fragments of: A. 11,12-Dehydro α-CPA (Compound 335); B: 3-Hydroxy-2-oxoCPA (compound 369), Table S1: List of *A. flavus* strains used in this study, Table S2: Characteristic fragments of oxindole CPA-type alkaloids.
