# Supplementary material for: Unravelling the Diversity of the Cyclopiazonic Acid Family of Mycotoxins in Aspergillus flavus by UHPLC Triple-TOF HRMS
Source: Toxins (Basel). 2017 Jan 13;9(1):35. doi: 10.3390/toxins9010035 (PMC5308267; doi:10.3390/toxins9010035)
Supplement: Supplementary file 1 [file toxins-09-00035-s001.pdf]

Supplementary Materials: Unravelling the Diversity of the Cyclopiazonic Acid Family of Mycotoxins in *Aspergillus flavus* by UHPLC Triple-TOF HRMS

Valdet Uka, Geromy G. Moore, Natalia Arroyo-Manzanares, Dashnor Nebija, Sarah De Saeger and José Diana Di Mavungu

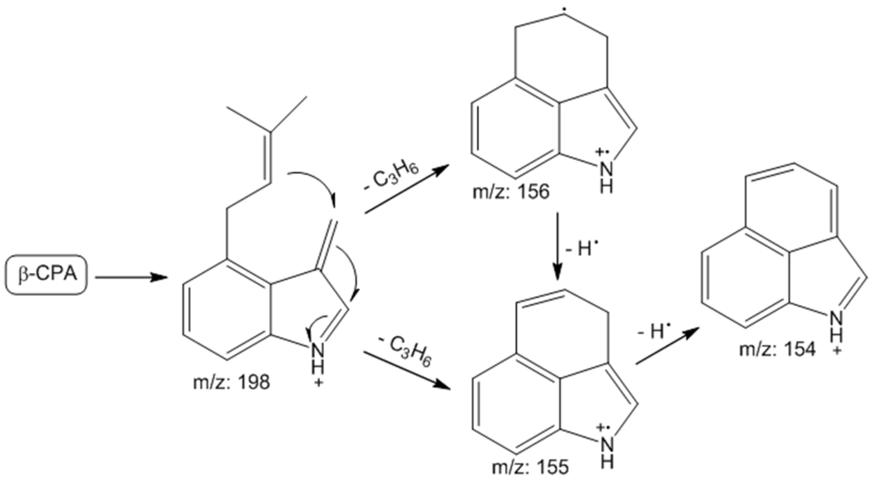

Figure S1. Cyclization mechanism of the ions at *m/z* 156, 155 and 154 in the fragmentation pathway of β-CPA.

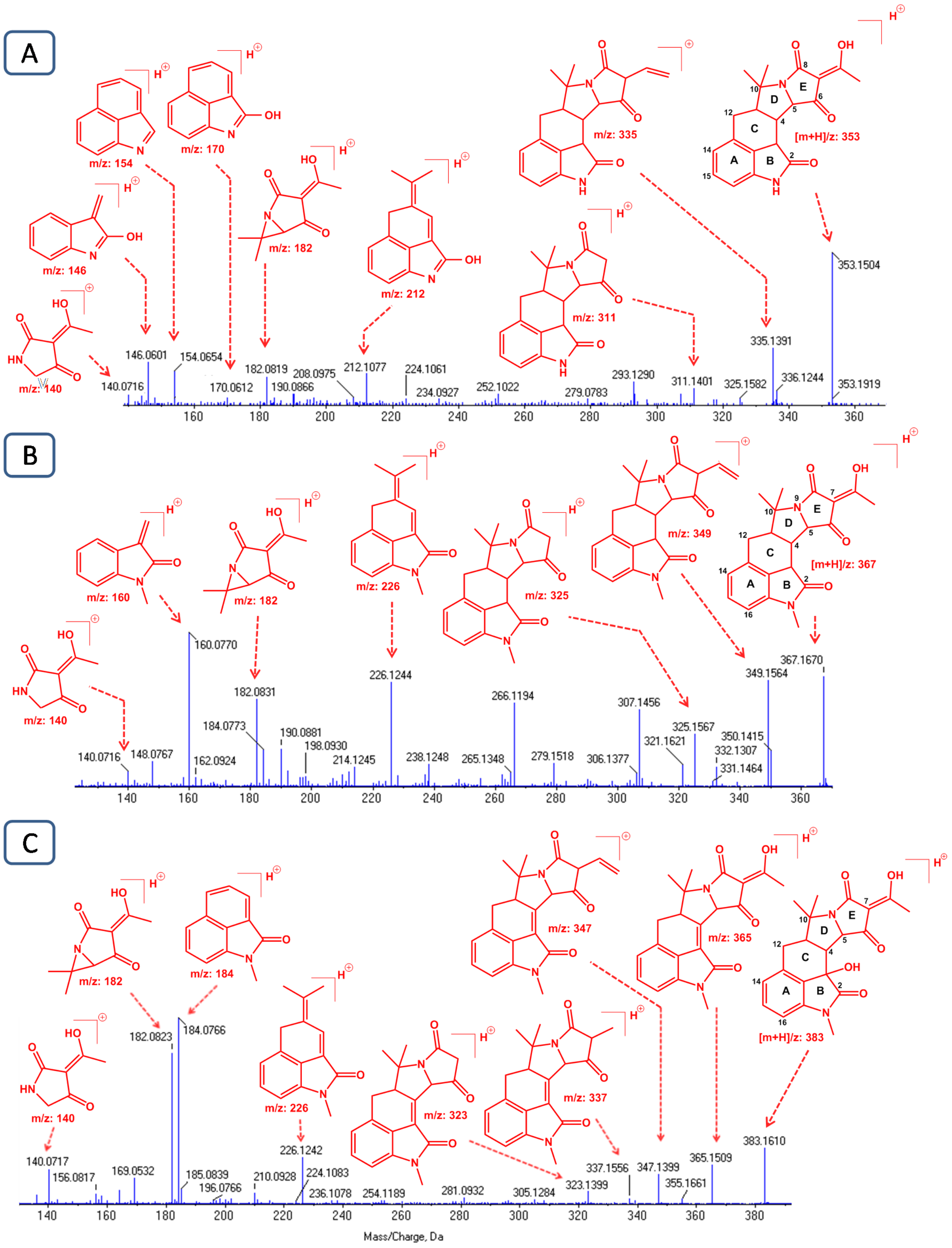

Figure S2. High resolution tandem mass spectrometry (HRMSMS) spectra and putative structural fragments of: (**A**) 2-oxo-CPA; (**B**) speradine A; (**C**) 3-hydroxy-speradine A. The HRMSMS spectra were acquired in IDA (information dependent acquisition) mode using a CE (collision energy) of 35 V with a collision energy spread (CES) of 15 V.

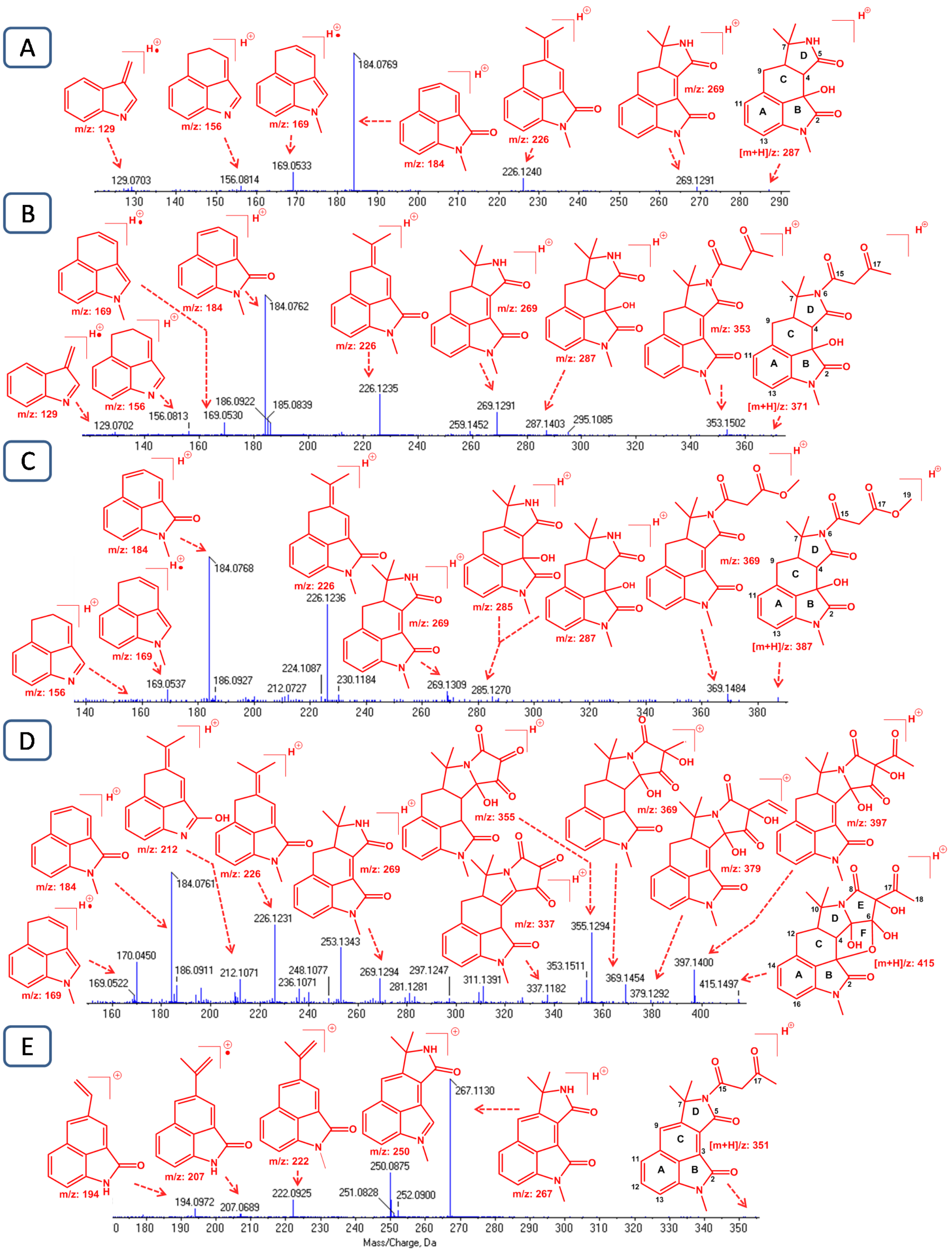

Figure S3. HRMSMS spectra and putative structural fragments of: (**A**) speradine B; (**B**) speradine C; (**C**) speradine D; (**D**) speradine F; (**E**) speradine H. The HRMSMS spectra were acquired in IDA (information dependent acquisition) mode using a CE (collision energy) of 35 V with a collision energy spread (CES) of 15 V.

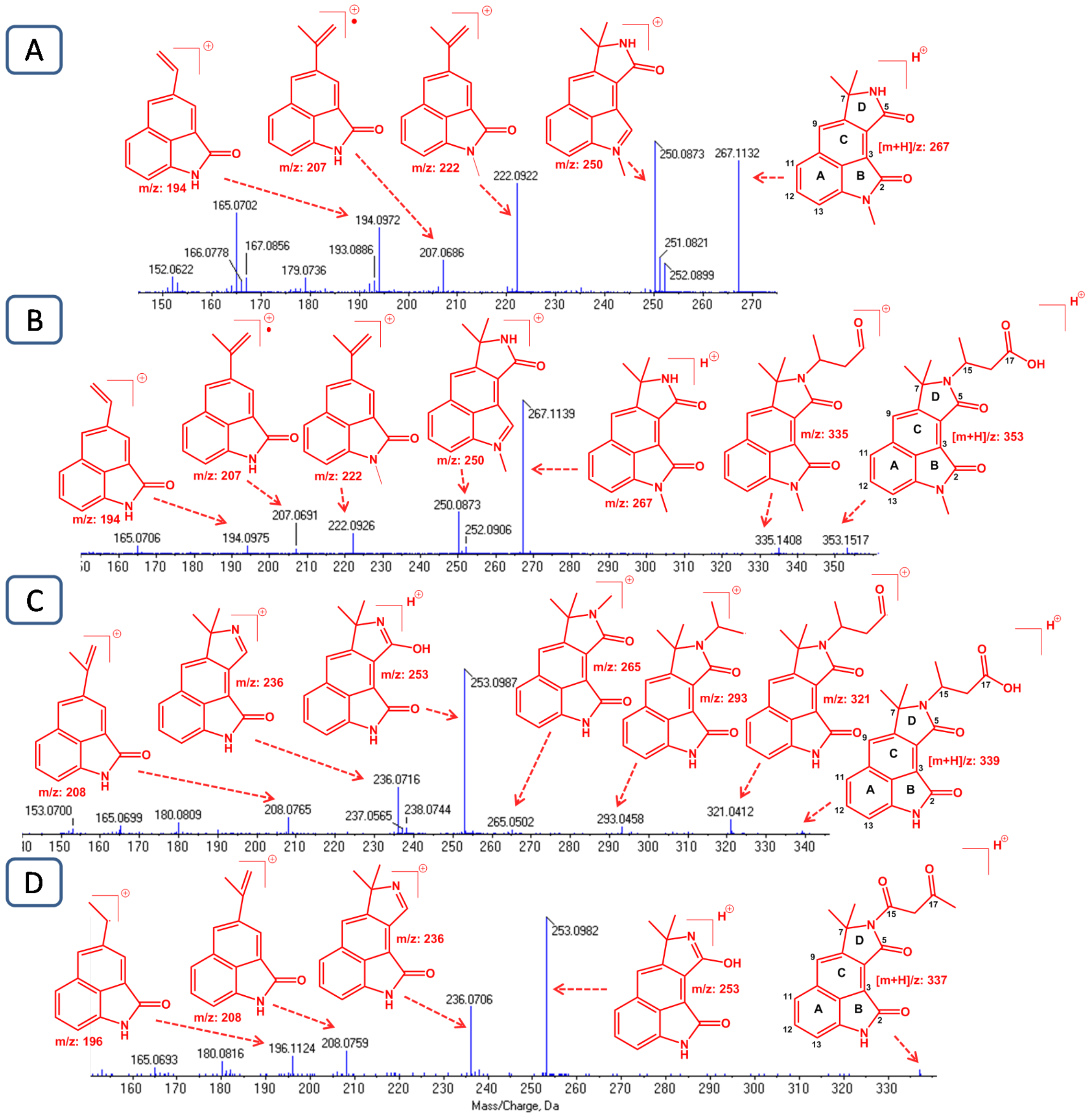

Figure S4. HRMSMS spectra and putative structural fragments of: (**A**) cyclopiamide A; (**B**) cyclopiamide B; (**C**) cyclopiamide C; (**D**) cyclopiamide D. The HRMSMS spectra were acquired in IDA (information dependent acquisition) mode using a CE (collision energy) of 35 V with a collision energy spread (CES) of 15 V.

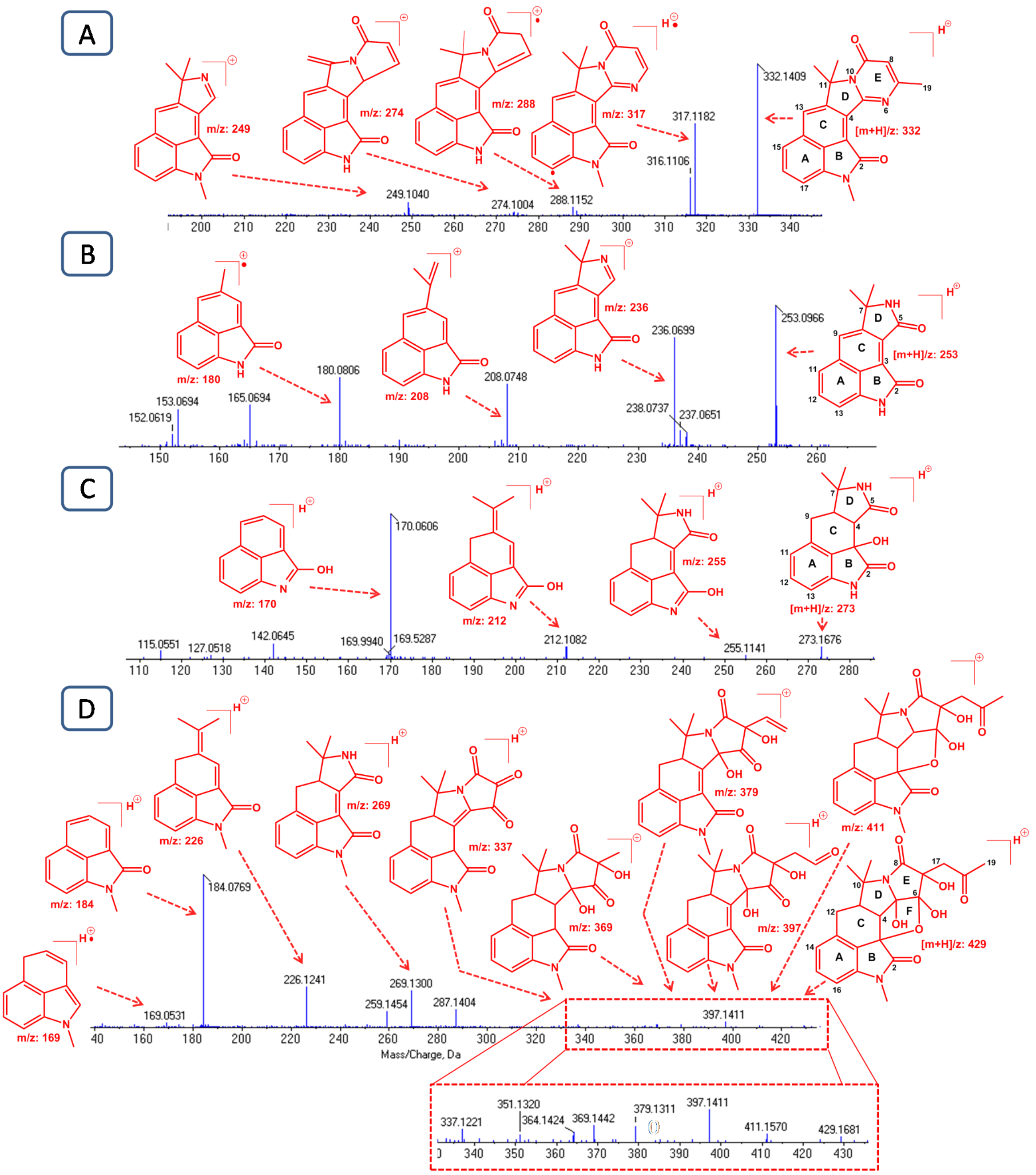

Figure S5. HRMSMS spectra and putative structural fragments of: (**A**) cyclopiamide E; (**B**) cyclopiamide F; (**C**) cyclopiamide G; (**D**) cyclopiamide J. The HRMSMS spectra were acquired in IDA (information dependent acquisition) mode using a CE (collision energy) of 35 V with a collision energy spread (CES) of 15 V.

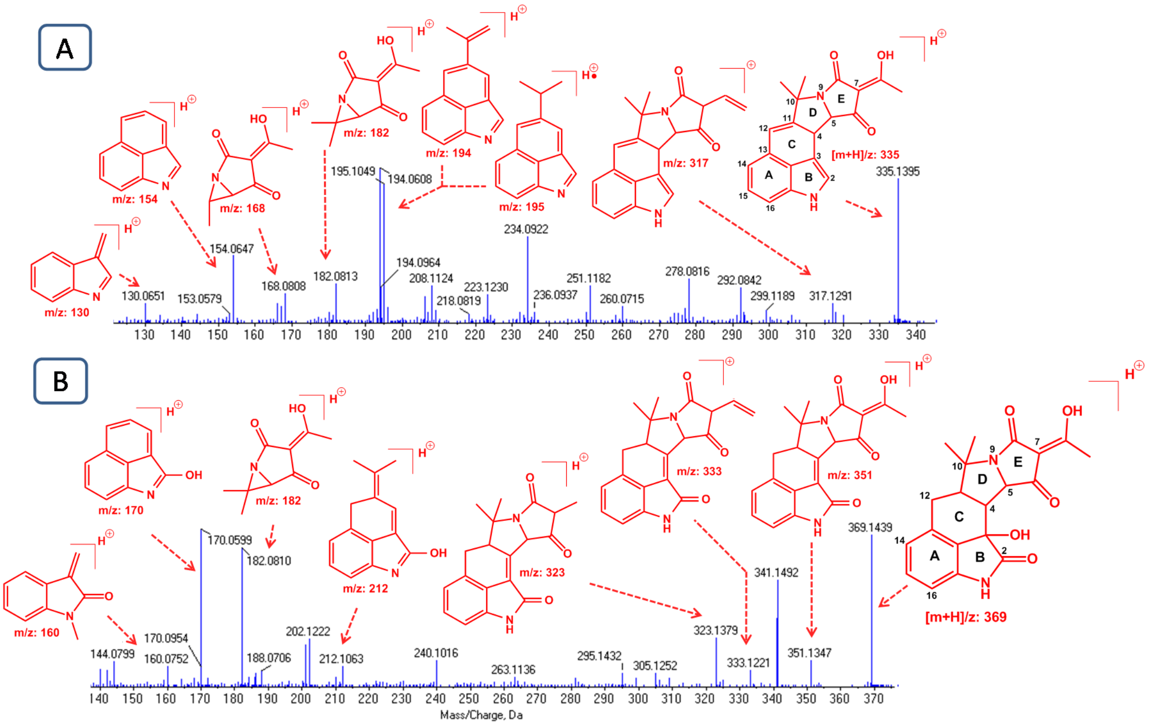

Figure S6. HRMSMS spectra and putative structural fragments of: (**A**) 11,12-Dehydro α-CPA (compound 335); (**B**) 3-Hydroxy-2-oxoCPA (compound 369). The HRMSMS spectra were acquired in IDA (information dependent acquisition) mode using a CE (collision energy) of 35 V with a collision energy spread (CES) of 15 V.

Table S1. List of *A. flavus* strains used in this study

SRRC ID	Other Designations	Source	Sclerotium morphotype [a]
0038	NRRL A-12268; ATCC 26938	turkey feed, Washington D.C.	S
0141	ATCC 24109; Pep-70-1hle	black pepper	S
0144	NRRL A-16464; SU25	cottonseed, Louisiana	U
0150	TR 955	cottonseed, Arizona	U
0151	TR UNK3	cottonseed, Arizona	S
0167	NRRL 3357; CBS128202; ATCC 200026	domestic peanut cotyledon	L
0283	NRRL 5918; SRRC 296	corn, Minnesota	S
0295	NRRL 3537; SRRC 284; ATCC 9643	shoe sole, Papua New Guinea	S
1000F	GH flavus #257	cottonseed	U
1006	012981-7	germinating cotton seed	U
1020	061181-10	cottonseed, Florence	U
1021	061281-5	cottonseed, Florence	S
1055	-	cotton boll, Arizona	U
1071	-	cottonseed, Arizona	U
1098	-	cottonseed, Arizona	U
1118	-	cottonseed, Arizona	U
1187	-	cottonseed, Arizona	U
1299	P. Cotty #12	soil, Arizona	S
1356	-	dried bacon, Croatia	L
1357	-	dried bacon, Croatia	S
1533 [b]	AF36; NRRL 18543	cottonseed, Arizona	L
1534 [b]	Afla-guard; NRRL 21882	peanut, Georgia	L
1540	BS07	Bayside, Texas	L
1541	CA 1	pistachio, California	S
1543	CA 3	pistachio, California	S
1544	CA 4	pistachio, California	S
1545	CA 5	pistachio, California	L
1547	CA 7	pistachio, California	L
1552	CA 12	pistachio, California	U
1553	CA 13	pistachio, California	S
1554	CA 14	pistachio, California	L
1557	CA 17	pistachio, California	S
1558	CA 18	pistachio, California	L
1559	CA 19	pistachio, California	L
1565	CA 26	pistachio, California	L
1566	CA 28	pistachio, California	S
1568	CA 32	pistachio, California	S
1571	CA 37	pistachio, California	S
1573	CA 39	pistachio, California	S/L
1574	CA 40	pistachio, California	L
1575	CA 41	pistachio, California	L
1576	CA 42	pistachio, California	S
1578	CA 44	pistachio, California	S
1591	SF-1	rain forest soil, Nigeria	S
1626	SF-32	cowpea, Nigeria	S
1637	SF-48	bread, Nigeria	S
2000	-	cottonseed, Arizona	U
2001	-	cottonseed, Arizona	L
2033	FER 2749	peanut rhizosphere, Australia	S
2035	FRR 2748	peanut, Australia	L
2114	ATCC 15546; FRR 3339; NRRL 6108	moldy wheat, Illinois	L
2115	VDR 15	sunflower seed, South Africa	L
2118	N-63-9	dried fish, Indonesia	L
2524	FC017; T-19	dead termites in China	L
2711	-	-	U

[a] L = large sclerotia (>400 μm); S = small sclerotia (<400 μm); U = unknown or non-sclerotial. [b] Commercially-available biopesticide. SRRC—Southern Regional Research Center, New Orleans, LA, USA; NRRL—National Center for Agricultural Utilization Research, Peoria, IL, USA; ATCC—American Type Culture Collection, Manassas, VA, USA; FRR—Food Research Laboratory, CSIRO, North Ryde, N.S.W., Australia; CBS—Centraalbureau voor Schimmelcultures, Utrecht, The Netherlands

Table S2. Characteristic fragments of oxindole CPA-type alkaloids

Oxindole subclass of CPA-type alkaloids		
**Chemical group**	**Metabolites**	**MS/MS fragments**
2-oxindoles with saturated ring C	2-oxo-CPA; cyclopiamide G	170, 212
2-oxindoles with unsaturated ring C	cyclopiamides C, D, F	253, 236, 208, 180, 165
N-methyl-2-oxindoles with saturated ring C	3-hydroxy-speradine A; speradines A-D, F; cyclopiamides H, J	269, 226, 184, 169, 156
N-methyl-2-oxindoles with unsaturated ring C	speradines E, H; cyclopiamides A, B, E	267, 250, 222, 207, 194

© 2017 by the authors. Submitted for possible open access publication under the terms and conditions of the Creative Commons Attribution (CC-BY) license (http://creativecommons.org/licenses/by/4.0/).
